# Advancing conventional guidelines in phytotoxicity studies assessing toxicant’s impact on seedling morphology and anatomy

**DOI:** 10.1038/s41598-025-92862-4

**Published:** 2025-07-02

**Authors:** Bárbara S. Diogo, Sara Rodrigues, Sara C. Antunes

**Affiliations:** 1 ICBAS, Instituto de Ciências Biomédicas de Abel Salazar, Universidade do Porto, Rua de Jorge Viterbo Ferreira, 228, 4050-313 Porto, Portugal; 2 CIMAR/CIIMAR LA, Centro Interdisciplinar de Investigação Marinha e Ambiental, Universidade do Porto, Terminal de Cruzeiros do Porto de Leixões, 4450-208 Matosinhos, Portugal; 3 FCUP, Departamento de Biologia, Faculdade de Ciências, Universidade do Porto, Rua do Campo Alegre s/n, 4169-007 Porto, Portugal

**Keywords:** Germination assays, Ecotoxicity, *Lactuca sativa*, Morphological and anatomical alterations, Plant sciences, Plant development

## Abstract

Studies on plant ecotoxicology focus essentially on growth and biochemical processes, often overlooking anatomical and morphological alterations that may occur post-germination. These changes, however, offer valuable insights into early environmental stress, enabling proactive intervention and mitigation strategies. Thus, this study aimed to identify and characterize morphological and anatomical alterations during early seedlings’ development, using a new Visual PhytoToxicity assessment (ViPTox) approach. This visual scoring system categorizes the alterations recorded into severity levels, offering a simple, reproducible method for assessing phytotoxicity based on observable changes in plant structure. A standard germination assay with *Lactuca sativa* was conducted using potassium dichromate (PD, reference compound) at 0.00, 100.5, 120.6, 144.7, 173.6, 208.3, and 250.0 mg/L. Standard endpoints, including germination rate, seedling size, and fresh and dry weight were evaluated alongside a novel ecotoxicological approach. Based on the seedling effects observed, a dichotomous key with a scoring system was defined using a classification range from 0 (normal seedling) to 10 (no germination - maximum damage), providing insights into the severity of observed alterations (e.g., absence of roots and/or leaves (score 9), chlorosis and necrosis (score 8), atrophy (score 7, 6, and 5), deformations (score 4 and 3), reduction of size (score 2 and 1)), in order to calculate the phyto-morphological damage (PMD). Considering the standard endpoints, no significant alterations were observed in *L. sativa* germination. However, a significant decrease in seedling size (> 20 %) and fresh weight (> 50 %) was observed, after exposure to the highest PD concentrations (173.6, 208.3, and 250.0 mg/L). Regarding the ViPTox approach, PMD was observed in all concentrations ≥ 120.6 mg/L of PD. Significant effects were observed even at lower PD concentrations (120.6 and 144.7 mg/L) where phyto-morphological damages (e.g., atrophy and deformations) were quantified, while standard endpoints were unaffected. ViPTox presents a reproducible, non-invasive, and cost-effective approach to evaluate seedling responses to environmental stress, complementing traditional assessment techniques while providing crucial insights that support proactive intervention and effective mitigation strategies.

## Introduction

Among the diversity of ecotoxicological assays available, bioassays with plants are less frequently used in comparison with assays carried out on animal models^1^, and even human cells^2,3^. However, several authors have already reported the advantages of using phytotoxicity assays on biomonitoring and investigating the toxicity of environmental pollutants, namely metals, pharmaceuticals, and pesticides^4–8^, as they provide a cost-effective, early-warning system to detect harmful effects on plant health. Plants are key indicators of ecosystem health, and assessing their response to environmental stressors helps understand pollutant impacts, inform regulatory decisions, and guide environmental management practices for sustainable ecosystems^9^. reported that, among over 6,000 studies in aquatic ecotoxicology (using animals, plants, and fungi), only 25 % involed plant species, highlighting a significant underrepresentation of plants in ecotoxicological research. Only in the last decades have plant-based ecotoxicological assays, such as germination assays, begun to be relatively employed for research studies (due to the sensitivity response)^9^. Seed germination assays^10^ are simple, very reproducible, and with low costs involved^3^. These assays have already been shown to be a good indicator of environmental samples toxicity evaluation^11,12^. They are validated bioassays^10,13^ that allow the evaluation of germination and root formation by monocotyledonous or dicotyledonous seeds, as ecotoxicological effects, after contact with a potentially toxic agent (e.g., effluents, pharmaceutical products, heavy metals)^9,12^.

Although there are already standardized protocols/guidelines for these assays^10,13^ and a list of species historically used in plant testing (e.g., *Lactuca sativa*, *Phaseolus vulgaris*, *Zea mays*, and *Allium cepa*), a reductive and simplistic evaluation approach is carried out, observing only parameters of germination and growth. Most phytotoxicity assays with seeds focus exclusively on germination, assessing whether seeds sprout and comparing results to a control. These assays calculate a germination index (GI) to indicate phytotoxicity levels: low GI values (e.g., < 75 %) suggest high phytotoxicity, while high values (e.g., > 85 %) indicate low or no toxicity^14^. Nevertheless, there are several non-standard parameters (e.g., test time, number of seeds per replicate) that remain variable, and which end up making a comparison between studies with the same species difficult^3,10^. Moreover, most studies on plants focus on the effects of various stresses on growth parameters (e.g., size and biomass), biochemical processes (e.g., antioxidant enzymes activities, and pigments content)^12,15^, ignoring the detrimental visual effects (e.g., chlorosis, necrosis, plant development abnormalities), also recommended by the guidelines^10^. Anatomical and morphological alterations or damage to plant tissue and/or cells (e.g., chlorosis, necrosis, atrophy) can also be used as indicators of phytotoxicity in plants^12,16^. Zhao et al.^17^, reported that Cr (100 to 400 µmol/L) affects plant morphology, and causes irreversible anatomical (e.g. decrease in root area) and ultrastructural changes (e.g. cell wall disorganization). However, the harmful effects on plant development and performance (e.g. inhibition of growth, low biomass accumulation, necrotic lesions, and chlorosis) can be considered nonspecific alterations, since similar effects were already recorded after exposure to different heavy metals (e.g., Pb, Cd, and Al), pharmaceuticals (e.g., antibiotics, and anti-inflammatories), and mixtures of compounds (e.g^12,18–20^).

The appearance of visual phytotoxic effects (e.g. necrosis, chlorosis, atrophy) and the occurrence of malformations in the different parts of the plants (leaves, roots, or both) can indicate the stress level^21^. Although these effects have been reported in some studies, their description and categorization are only rough and superficial, and the illustrations are insufficient^21^. However, these observations make the comparison of results difficult since the extent of the damage observed is not quantified. Indeed, other scientific areas (e.g., histopathology) have used a scoring system to correlate and quantify the changes observed in different organisms/organs following exposure to different stressors^22–24^. Firstly, this approach evaluates changes qualitatively and then calculates pathological indices, based on the extent and the severity degree of the damage observed in the analyzed sample. However, this sensitive method has yet to be applied to complement the study of ecotoxicological effects in plants (e.g., phytotoxicity).

Thus, this study aims to describe and categorize morphological and anatomical alterations in the early plant development of *Lactuca sativa*, developing and validating a scoring system (Ecotoxicological approach based on Visual PhytoToxicity assessment - ViPTox) that can be used as a complementary analysis of the phytotoxicity studies. To achieve the objective of this study, germination assays with *Lactuca sativa* were carried out using a reference substance, potassium dichromate^13,25–27^, and a set of standard and non-standard endpoints were evaluated. Given its visual-based scoring system, ViPTox can be integrated into the current OECD guidelines to enhance the detection of early-stage phytotoxicity, particularly for pollutants that cause subtle morphological damage. Furthermore, for regulatory purposes, ViPTox can be adopted as an additional tool to provide more comprehensive data on plant toxicity, aligning with regulatory frameworks that aim for more sensitive and ecologically relevant testing.

## Materials and methods

### Potassium dichromate

Potassium dichromate (PD) (Cr_2_K_2_O_7_; molecular weight 294.18 g/mol), is used as a reference substance in aquatic and terrestrial toxicology assays, due to its well-documented toxicological profile and its content of hexavalent chromium (Cr⁶⁺), a highly toxic and environmentally relevant compound commonly found in industrial areas and contaminated water bodies^13,25–28^. PD was acquired at Sigma Aldrich (CAS 7778-50-9), and a stock solution of 250.0 mg PD/L was prepared with sterile deionized water. To conduct the germination assay, 7 concentrations [0.00 (control), 100.5, 120.6, 144.7, 173.6, 208.3, 250.0 mg PD/L] were defined, based on the phytotoxicity reported in the literature [EC_50_ = 133.24 (CI95% =113.33–178.36) mg PD/L)]^29^.

### Germination assay

The germination assay was conducted according to OECD (2006) guidelines, with few adaptations described in^12^. For the germination assay, *Lactuca sativa* seeds (Vilmorin, France) were sterilized by a bath in 5 % sodium hypochlorite (NaClO) for 5 min, followed by 3 washes in sterile deionized water. After being sterilized, in a flow chamber, 10 seeds were placed in each sterile square Petri dish containing solid Hoagland’s solution^30^, with 1.5 % agar and 10 mL of each PD concentration. For the control group, 10 mL of sterile deionized water was added to the Petri dish. For each treatment, three replicates were prepared (3 square plates, each one with 10 seeds). All the Petri dishes were placed vertically in a climate chamber, under controlled conditions of temperature (20 ± 2 ºC), photoperiod (16 h^L^:8 h^D^), and luminosity (~ 6000 lx). At the end of the exposure period (fourteen days after 50 % o seedling emergence in the control group), the percentage of seed emergence was evaluated. The seedlings were used for biometric evaluations, namely aerial part, roots size (cm), and fresh and dry biomass (g)^10^. Before that, all seedlings (30 replicates per concentration) were photographed for subsequent observation of the morphologic nonspecific alterations in early plant development (e.g., damage/deformations, chlorosis, wilting). To minimize subjectivity, a blind scoring of the key findings was independently conducted by three different observers. Previous studies reported some of these morphological alterations (e.g^12^), however, the severity was not evaluated. Thus, based on the new approach ViPTox, the results were analyzed to categorize the different morphological alterations and consider their degree of severity (each seedling/replicate had a toxicity score assigned). The phyto-morphological damage was calculated, per each concentration tested, according to:

$${\text{PMD }} = \frac{{\sum \left( {{\text{IS}}} \right)}}{n}$$

Where, PMD = Phyto-Morphological Damage; IS = individual score obtained in each seedling (replicate); n = total number of seedlings (replicates).

**Fig. 1:**
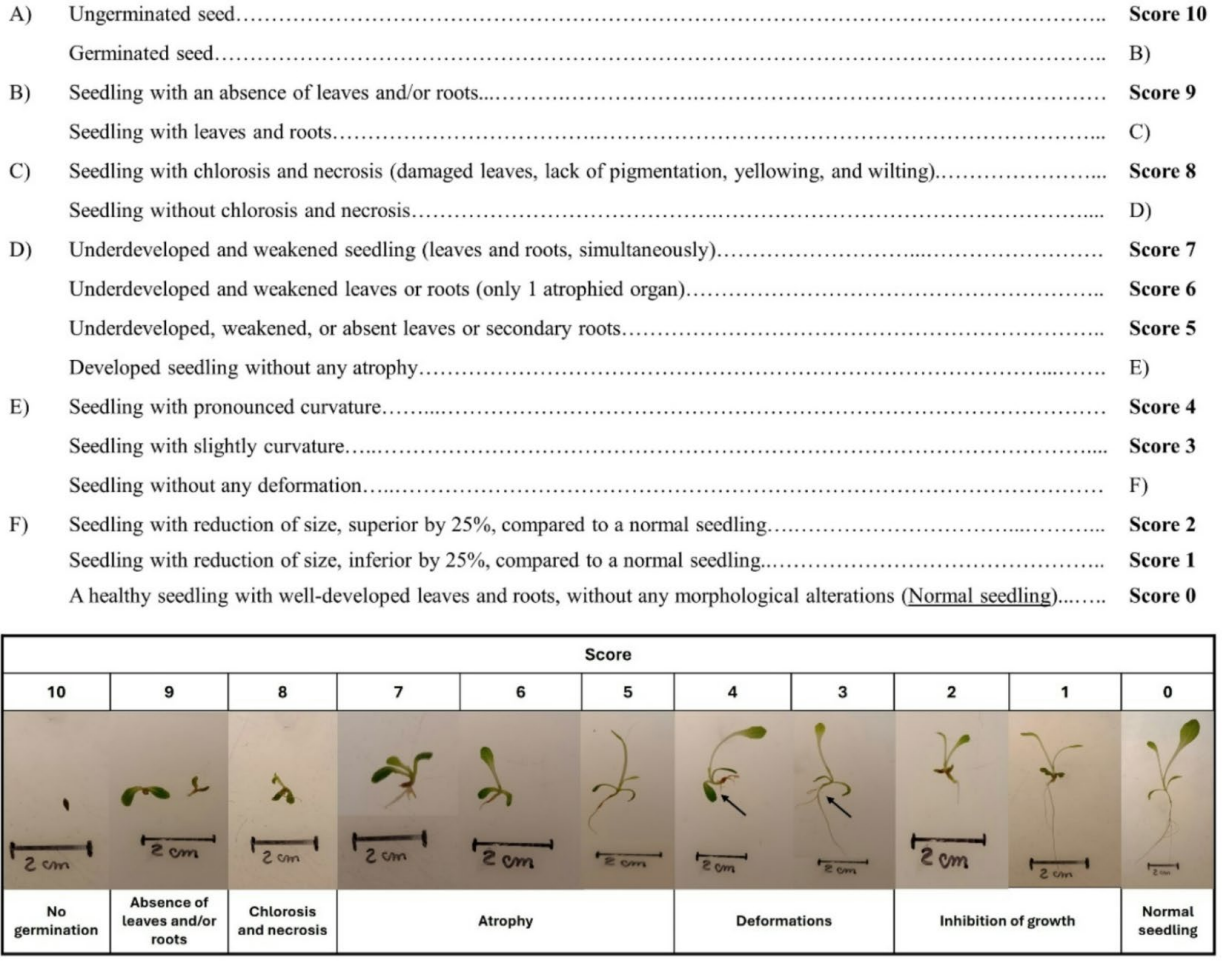
Visual PhytoToxicity assessment (ViPTox) – dichotomous key with a scoring system that allows us to identify and categorize the different morphological and anatomical alterations observed. Toxicity scores range from 0 (normal seedling) to 10 (maximum damage, no germination).

The ViPTox approach uses a dichotomous key with a toxicity scale to identify and categorize morphological and anatomical alterations (Fig. 1). This system classifies damage based on severity and quantifies the number of seedlings affected in each test condition, assigning a toxicity score ranging from 0 (no damage) to 10 (higher damage). Each step of the dichotomous key is accompanied by descriptions and visual images that lead to the individual damage score (Fig. 1).

### Statistical analysis

All the evaluated endpoints (germination, size, fresh and dry biomass, and PMD) were checked for normality by the Shapiro-Wilk test and for homogeneity of variances by Levene’s test. A one-way analysis of variance (ANOVA) was performed, followed by a Dunnett’s test to discriminate differences between the PD concentrations and the control treatment. All the statistical analyses were performed using the software SPSS Statistics v29, considering a significance level of α = 0.05.

## Results and discussion

### OECD requirements: germination and growth

To comply with the requirements of guideline nº 208 ^10^, the effects of PD exposure on the germination assay with *L. sativa* were assessed, including the parameters emergence percentage, size of aerial part and roots, and fresh and dry biomass, as presented in Table 1. After exposure to PD no significant effects in the percentage of germination (F_[6, 20]_ = 0.833, *p* = 0.564), fresh biomass (F_[6, 20]_ = 2.162, *p* = 0.110) and dry biomass (F_[6, 20]_ = 0.567, *p* = 0.750) of the roots, and dry biomass of the aerial part (F_[6, 20]_ = 2.343, *p* = 0.089) were observed. Nonetheless, a significant decrease in the aerial part size of *L. sativa* (F_[6, 20]_ = 10.240, *p* < 0.001) was observed after exposure to the three highest concentrations of PD (173.6, 208.3, and 250.0 mg/L). On the other hand, only the highest concentration (250.0 mg PD/L) was able to decrease significantly the root size (F_[6, 20]_ = 7.191, *p* < 0.001), and fresh biomass of the aerial part (F_[6, 20]_ = 5.048, *p* < 0.001). Peduto et al.^31^ reported that 50 mg PD/L can be moderately toxic for *Lepidium sativum*,* Eruca sativa*, and *Cucumis sativus*, while is toxic for *Sinapis alba* and *Lactuca sativa*, considering the effects in germination after 7 days of exposure. Silva and Mattiolo^14^ evaluated the effect of PD (2.5, 5, 10, 20, and 50 mg/L) on the germination of *L. sativa* and did not observe significant effects in standard endpoints. However, although 50 mg PD/L was not enough to affect the germination of *L. sativa* seeds, this concentration caused a significant decrease in root growth^14^. Although the here-present study also evaluated the effect of PD on the germination and growth of *L. sativa*, direct comparisons with the findings of Silva and Mattiolo^14^ should be made carefully due to the differences in assay conditions, particularly, the exposure time (5 days vs. 14 days), the substrate used (qualitative filter paper vs. agar medium), and the time to germination (5 days vs. 3 days), which could affect the overall outcome.

**Table 1:** Percentage of germination (%), size (cm), fresh and dry biomass (g) observed in the aerial part and roots of *Lactuca sativa* after exposure to a range concentrations of potassium dichromate (mg/L).

Potassium dichromate (mg/L)	Germination (%)	Size (cm)		Fresh Biomass (g)		Dry Biomass (g)	
		Aerial part	Roots	Aerial part	Roots	Aerial part	Roots
0.0	100.0	4.57 ± 0.11	4.04 ± 1.20	223.8 ± 20.8	17.5 ± 1.2	10.7 ± 1.1	4.03 ± 0.23
100.5	95.0	4.67 ± 0.10	4.96 ± 0.46	270.6 ± 15.0	16.9 ± 0.7	20.3 ± 1.5	4.03 ± 0.88
120.6	97.5	4.54 ± 0.36	3.67 ± 0.70	189.7 ± 46.0	14.0 ± 2.6	21.1 ± 5.2	4.23 ± 1.49
144.7	100.0	4.06 ± 0.07	4.32 ± 0.28	212.3 ± 12.6	14.9 ± 0.6	20.2 ± 1.4	5.10 ± 0.59
173.6	100.0	3.86 ± 0.22*	3.01 ± 0.33	164.9 ± 15.1	11.2 ± 3.3	15.0 ± 1.4	3.57 ± 0.38
208.3	100.0	3.61 ± 0.21*	2.45 ± 0.35	132.2 ± 21.9	10.3 ± 1.6	14.9 ± 0.9	3.60 ± 0.21
250.0	97.5	3.76 ± 0.49*	1.74 ± 0.24*	112.2 ± 23.1*	12.4 ± 1.4	15.2 ± 3.1	3.57 ± 0.35

*Significant differences when compared to the control group (0.0 mg/L potassium dichromate) (Dunnett test, *p* < 0.05).

### ViPTox approach: a new visual phytotoxicity analysis

As proposed by guideline nº 208^10^ in the observations section, other studies already used a scoring system for performing qualitative and quantitative visual alterations on plants [e.g^32,33^). Hamill et al.^32^ studied the performance of herbicides, using a scoring system between 0 and 10, where 0 represents the low herbicide performance (higher plant growth) and 10 the maximum performance (absence of plant growth). However, neither other studies nor the OECD guideline provides tools or methodologies to perform careful visual analysis to identify the morphological and anatomical alterations (e.g., inhibition of growth, deformations, necrotic lesions) in aerial parts and roots of plants. Existing indices (e.g., germination indices) typically focus on basic endpoints, such as germination rate and seedling size, which overlook critical changes in plant morphology and anatomy, that may occur post-germination. These indices cannot differentiate between degrees of damage capture sub-lethal effects, or account for responses beyond the seed’s nutrient reserves making them insufficient for comprehensive phytotoxicity assessments. ViPTox addresses these gaps by incorporating a detailed scoring system that evaluates, both the severity and extent of morphological alterations, providing a more nuanced understanding of toxic effects. Its dichotomous key enhances precision and reduces subjectivity, offering a broader and more reliable framework for phytotoxicological studies compared to the traditional GI methods. Thus, to address these gaps, in the present study after *L. sativa* exposure to PD, a new evaluation of the toxicity scale was proposed to categorize and quantify morphological and anatomical damage, by visual phytotoxicity analysis - ViPTox (Fig. 1).

Morphological alterations can affect plant development, but their effects on plant performance and ecosystem roles depend on the severity of the observed changes. Unlike previous studies, which often describe visual effects more subjectively, ViPTox provides a standardized scoring system that ensures more consistent, reproducible, and reliable assessments. This innovation allows for a more precise evaluation of phytotoxicity, particularly by categorizing and quantifying the severity of morphological and anatomical alterations, which facilitates comparisons across studies and offers a deeper understanding of the effects of a wide range of contaminants, like PD. Non-germination of seeds is considered the most evident effect of toxic compounds on plants^20^, with the highest level of the toxic effect being considered on our severity scale (score 10). Indeed, this effect is particularly severe and completely affects plant development. By including this extreme case in the ViPTox scale, we ensure a comprehensive evaluation of plant stress, from minor alterations to complete failure in germination.

Morphological alterations in plants, including leaf deformities/injuries or root disruption (scores 8 and 9), can significantly be associated with a disruption in metabolic and physiological pathways crucial for survival and growth^12,34^. Furthermore, atrophies (scores 5, 6, and 7) or deformities (scores 3 and 4), often result from the weakening of a plant’s immune system, leaving it more susceptible to diseases and pest attacks^35^. All these changes interfere with different processes such as photosynthesis, water balance, nutrient absorption, and hormonal regulation^34,36^, compromising the plant’s ability to thrive^37^. Consequently, these disruptions can have severe consequences for plant development and growth^12,37^, emphasizing the importance of addressing the impacts of morphological damage in plants.

### Phyto-morphological damage (PMD)

**Fig. 2:**
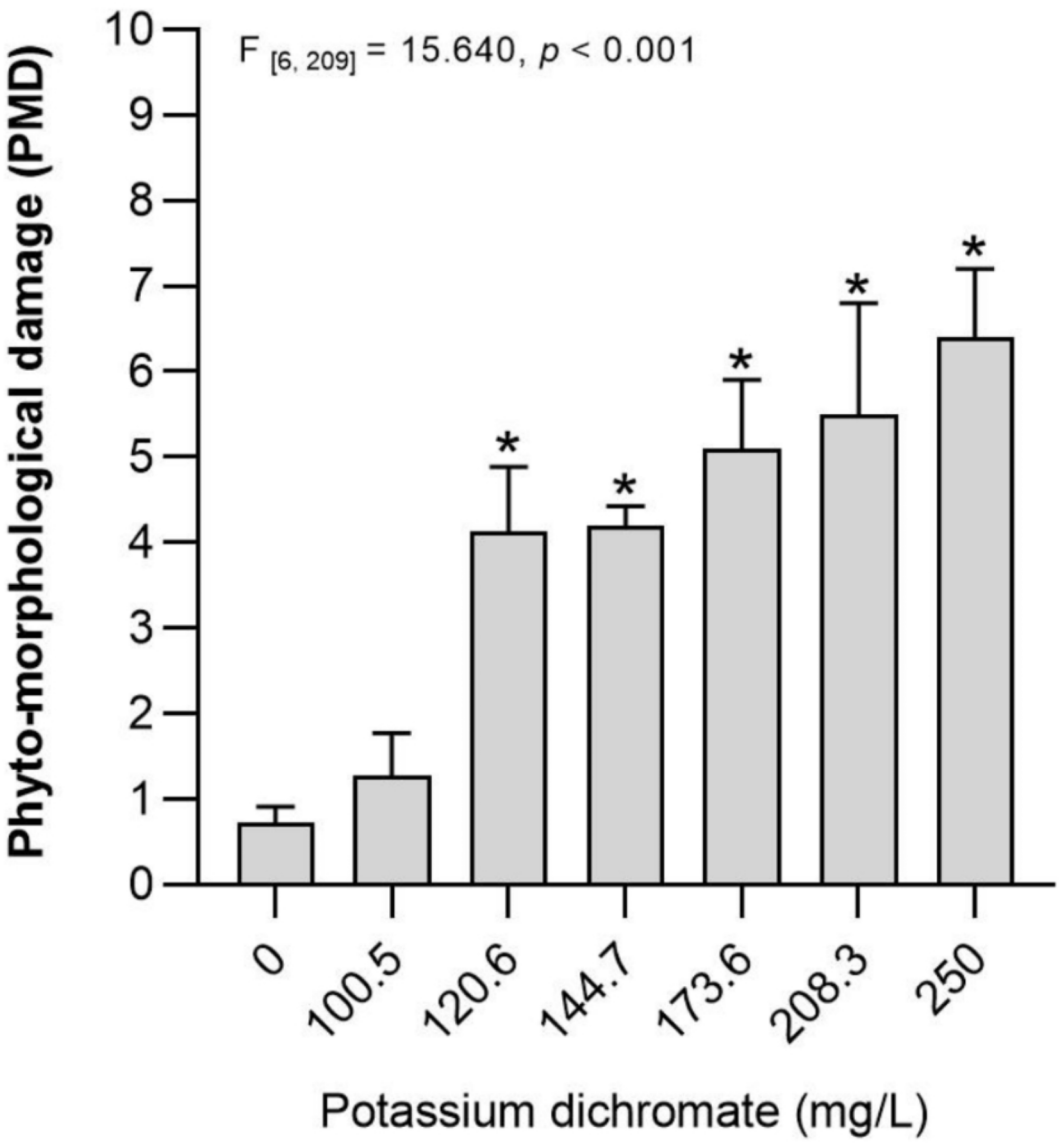
Phyto-morphological damage (PMD) [average (*n* = 30) ± standard error] obtained in *Lactuca sativa* after 14 days exposure to a range of concentrations of potassium dichromate (mg/L, reference substance). *Stands for discriminating significant differences between the control group (Dunnet *p* < 0.05).

Figure 2 represents the phyto-morphological damage observed in *L. sativa* after exposure to a range of concentrations of PD. Considering the results obtained, significant phyto-morphological damage compared to the control (without PD) was observed after exposure to concentrations up to 120.6 mg PD/L. PD is considered a Chromium VI compound [since it consists of hexavalent chromium (Cr (VI)]. Thus, most studies carried out with PD use this compound as a source of the heavy metal Cr (VI) and study the toxicity of Cr (VI) in the form of PD^13,38^. Cr (VI) is considered highly toxic to plants, being able to significantly reduce root growth and overall development^39^. It is also responsible for causing morphological changes (e.g., chlorosis and necrosis in leaves), since the different essential metabolic processes were affected (e.g., inhibiting photosynthesis)^40,41^. Zulfiqar et al.^42^ reported that stress caused by Cr compounds damages the morphophysiological attributes of plants and can be associated with the reduced membrane stability of plants (due to reactive oxygen species accumulation). Furthermore, these authors identified several consequences in morphological and biochemical parameters in plants exposed to Cr, namely a decrease in seed germination, plant biomass, and photosynthesis efficiency, and an increase in root deformations^42^.

The here-present results also showed that with the increase of PD concentrations, the seedling damage observed increases, being the higher PMD obtained after exposure to 250 mg/L of PD (~ score 7; Fig. 2). The OECD guideline parameters allow us to observe that only higher concentrations (up to 173.6 mg/L of PD) cause significant alterations in *L. sativa* seedlings (effects in size and fresh biomass of aerial part - Table 1). However, the ViPTox approach with PMD quantification demonstrated that lower concentrations (120.6 and 144.7 mg/L) also cause significant morphological alterations (Fig. 2), such as atrophy (scores 5, 6, and 7), and deformations (scores 3 and 4) enhancing the plant growth infeasibility. Several studies already showed that different compounds, or natural samples, caused effects on the biometric measures such as size and weight of *L. sativa* (e.g^14,31^), however, studies that reported and quantify the morphological alterations by phytotoxic visual analysis are scarce. These alterations can include different anomalies (e.g., impaired growth and development, chlorosis, necrosis, and wilting), and should not be ignored. The intensity and severity of visual alterations in leaves and roots (or both simultaneously) can be used as a sensible indicator of stress level, as demonstrated in the present study (Fig. 1 – score 9). Diogo et al.^12^ studied the effects of elutriates from coal mining wastes in *L. sativa* and proved that the morphological alterations (e.g., reduced growth, deformations, atrophy) can be a sensitive parameter to evaluate the phytotoxicity in plants. Moreover, these authors proposed a relationship between morphological damage observed and disturbed metabolic and physiological pathways [e.g., significant changes in antioxidant defense enzymes and pigment content, and a significant increase in malondialdehyde (MDA) content]. In our study, similar morphological alterations were observed, and these changes likely reflect underlying biochemical disturbances, such as oxidative stress or disruptions in metabolic pathways. Thus, if the stress is severe and/or prolonged, morphological alterations in leaves and/or roots can occur, which can be easily detected through visual analysis, and related to biochemical and/or physiological variations (e.g., MDA and pigments content).

Plants play crucial roles in various ecological processes (e.g. nutrient cycling supporting processes, soil carbon storage, pollination), and any changes in plants’ structure can disrupt these important interactions, affecting biodiversity and ecosystem stability^43^. Identifying and categorizing different morphological and anatomical alterations in plants, as well as assessing their intensity and severity, are essential approaches in ecological and toxicological research. These methods help us to understand the impact of environmental stressors on plant health and ecosystem dynamics. With ViPTox assessment, researchers can quantify the stress levels, and evaluate ecosystem resilience. This comprehensive approach also elucidates plants’ responses to various stresses, encompassing pollutants and climate change^44^, thereby enhancing our comprehension on the interplay between environmental factors, plant reactions, their adaptative responses, and broader ecological consequences. Additionally, the ViPTox approach showed to have great potential for use in environmental monitoring and phytoremediation studies. By assessing the morphological and anatomical alterations in plants exposed to environmental contaminants, can act as an early indicator of ecological stress and provide valuable insights into ecosystem health. In phytoremediation, ViPTox can be used to monitor the health and stress levels of plants utilized for contaminant removal, providing insights into the effectiveness of these processes and their impact on ecosystem health.

## Conclusions

This study introduced a new approach, the Visual PhytoToxicity assessment (ViPTox), by employing a dichotomous key (considering the severity and extent of alterations), with a simple scoring system (i.e. toxicity scale). ViPTox approach allows the standardized assessment of seedling responses to environmental stressors, quickly, non-invasive, and cost-effective. Furthermore, this approach is initiated from seedlings representing the pivotal early stages of plant development, enabling the deployment of targeted mitigation strategies at this critical juncture. This proactive measure holds the potential to avert more profound damage during subsequent stages of plant development. The results obtained for PD exposure showed that the ViPTox approach detected significant alterations in seedlings (such as atrophy and deformations) before the OECD guideline parameters. Thus, the ViPTox approach with germination assays and the evaluation of standard endpoints (e.g., growth and weight) demonstrated that can be used as a sensitive indicator of stress/toxicity. To fully realize its potential in regulatory frameworks, the ViPTox approach would need to be validated through comparative and inter-laboratory studies, ensuring its reliability and reproducibility across different species and environmental conditions. Once this optimization, ViPTox could serve as a complementary tool in phytotoxicity assessments, providing more comprehensive and ecologically relevant data on the toxic effects of environmental contaminants on plants. Understanding morphological and anatomical alterations in plants provides crucial insights into the early signs of environmental stress, allowing for proactive intervention and mitigation strategies. Incorporating AI models with the ViPTox approach presents an exciting opportunity to enhance its precision and scalability, enabling automated identification and classification of morphological damage. Furthermore, it offers a standardized framework for assessment and comparison in future studies, facilitating the quantifications of stress levels and predicting biochemical changes in plants. In essence, ViPTox represents an essential tool in plant studies, contributing to the advancement of our knowledge of plant-environment interactions, while the integration of AI expands its utility in ecotoxicological assessments and environmental monitoring.

## Data Availability

All the data produced in this study are presented in the manuscript.
